# Submandibular Ectopic Thymic Mass in a 6-Month-Old Infant

**DOI:** 10.1055/s-0042-1751055

**Published:** 2024-07-11

**Authors:** Jordan Fenner, Israel Saramago, Jorge Oldan, Mitchel Muhleman

**Affiliations:** 1 Department of Radiology, University of North Carolina School of Medicine, Chapel Hill, North Carolina, United States; 2 Department of Molecular Imaging and Therapeutics, University of North Carolina School of Medicine, Chapel Hill, North Carolina, United States

**Keywords:** ectopic thymic tissue, submandibular mass, F-18 FDG PET/CT

## Abstract

Infant ectopic cervical thymus is a relatively uncommon diagnosis and, in many cases, subclinical. If not subclinical, it may present as a palpable swelling or with compressive symptoms (i.e., stridor or dysphagia). Standard radiologic workup includes an ultrasound followed by magnetic resonance imaging (MRI) with tissue sampling if the ultrasound is indeterminate. In this case, an incidental submandibular mass was noted on a noncontrast MRI for seizures in a 6-month-old male infant. A radiologic and pathologic workup was performed for evaluation. However, this case is unique as fluorodeoxyglucose positron emission tomography/computed tomography was also utilized to potentially aid in the establishment of a diagnosis.

A 6-month-old male infant presented with a right submandibular mass of unknown origin, incidentally found on noncontrast magnetic resonance imaging (MRI) for seizure evaluation. Ultrasound of the submandibular mass demonstrates a mildly spongiform, mildly heterogeneous, hypoechoic internal architecture on sagittal ( Fig. 1A ) and transverse views ( Fig. 1B ) with internal vascularity on transverse view ( Fig. 1C ). While a review of the literature show these findings could be consistent with ectopic thymic tissue, which has been characterized as a well-defined lesion with linear echogenic septa with corresponding vascularity and hypoechoic rims, similar to normal thymus, 1 2 thymic tissue has unique features and it is sometimes difficult to differentiate ectopic thymic tissue from other neck pathologies. 3 In this study the findings were reported as nonspecific and suggested further evaluation with a contrast-enhanced MRI and tissue sampling.

**Fig. 1:**
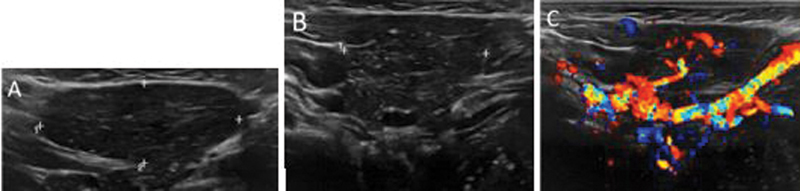
Ultrasound of the submandibular mass demonstrates a mildly spongiform, mildly heterogeneous, hypoechoic internal architecture on sagittal ( **A** ) and transverse views ( **B** ) with internal vascularity on transverse view ( **C** ).

On the follow-up contrast-enhanced MRI of the neck, the mass ( *arrow* ) measures 2.3 × 3.6 × 2.1 cm on MRI of the neck and is homogeneous and isointense to muscle ( *arrowhead* ) on axial T1-weighted image ( Fig. 2A ), and hyperintense to muscle ( *arrowhead* ) on axial T2-weighted image ( Fig. 2B ). These findings were reported as nonspecific, with differentials including conglomerate lymphadenopathy related to a lymphoproliferative process or a mesenchymal tumor such as a sarcoma. Although uncommon, the differential could have included normal thymic tissue in an ectopic cervical location along the expected developmental path from the third and fourth branchial pouch. 1 4 5 The fine needle aspirate with flow cytometry was performed showing mature T-cells, but neoplasm (versus benign thymic tissue) was not excluded. Due to the inconclusive MRI report and the biopsy unable to exclude malignancy, a contrast-enhanced fluorodeoxyglucose positron emission tomography/computed tomography (F- ^18^ FDG PET/CT) was ordered for further evaluation.

**Fig. 2:**
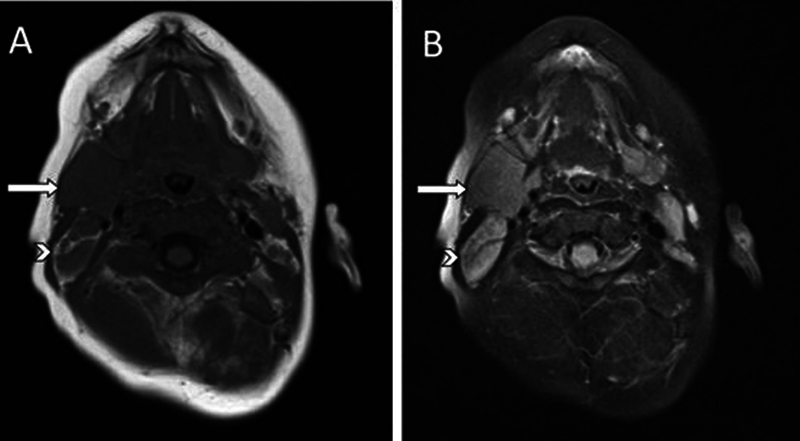
The mass ( *arrow* ) measures 2.3 × 3.6 × 2.1 cm and is homogeneous and isointense to muscle ( *arrowhead* ) on axial T1-weighted image ( **A** ), and hyperintense to muscle ( *arrowhead* ) on axial T2-weighted image ( **B** ).

Intravenous contrast-enhanced CT neck ( Fig. 3A, B ) was obtained concurrently with the PET, which demonstrates a nonenhancing mass in the right submandibular space ( *arrow* ).

**Fig. 3:**
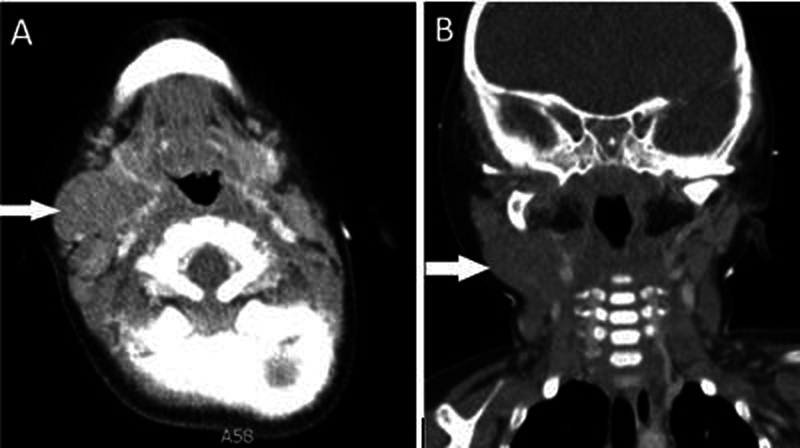
Intravenous contrast-enhanced CT neck ( **A, B** ) was obtained concurrently with the PET, which demonstrates a nonenhancing mass in the right submandibular space ( *arrow* ).

The PET/CT demonstrates a mildly FDG-avid submandibular mass ( *arrow* ) (maximum standardized uptake value [SUVmax] 2.4) as seen in both the anterior maximum intensity projection and coronal views ( Fig. 4A, B ), with similar homogeneous uptake and soft tissue density to the mediastinal thymus ( *arrowhead* ) (SUVmax 2.5) ( Fig. 4C, D ). There are prominent but nonavid right level II B-lymph nodes. Ectopic thymic tissue frequently demonstrates mild FDG uptake, similar to normal thymic tissue, as in this case. It has been suggested that the intensity of FDG uptake may aid in differentiating between various thymus lesions. Lesions such as thymic carcinoma, thymoma, and thymic hyperplasia may all demonstrate varying degrees of uptake, with significantly increased uptake in thymic carcinoma compared with thymomas or the normal thymus. 6 7 Normal thymic uptake is also lower than other metabolically active processes such as T-cell predominant lymphoma. 1 6 8 The mild uptake of the cervical mass (greater than fat and muscle but equal to the thymus), lack of necrosis, and low potential disease extent helped point toward a benign etiology.

**Fig. 4:**
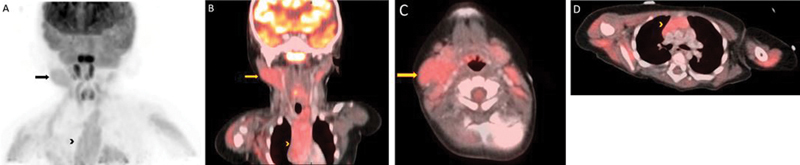
The PET/CT demonstrates a mildly FDG-avid submandibular mass ( *arrow* ) (SUVmax 2.4) as seen in both the anterior maximum intensity projection and coronal views ( **A, B** ), with similar homogeneous uptake and soft tissue density to the mediastinal thymus ( *arrowhead* ) (SUVmax 2.5) ( **C, D** ).

Recently, there has been a migration to conservative treatment in normal nonsymptomatic ectopic thymic tissue: imaging alone versus surgical biopsy or MRI with fine needle aspiration (FNA). The conservative approach requires confidence in the initial identification of benign ectopic thymic tissue and serial imaging with preparation for FNA or excisional biopsy in the setting of characteristic changes. 9 In this particular case, an open excisional biopsy was indicated due to the neck mass contributing to the interval development and continued contribution to plagiocephaly. The final surgical pathology confirmed the diagnosis of benign thymic tissue, negative for malignancy. Ectopic thymic tissue often presents asymptomatically in childhood, and due to its benignity it is important to keep in mind when presented with a cervical mass in a pediatric patient. 1 10 11 F-18 FDG PET/CT's potential to aid in distinguishing benign versus malignant etiologies, based on metabolic activity rather than anatomical characteristics, is well documented. This case illustrates the potential added benefit of F-18 FDG PET/CT in the assessment of cervical masses of unknown origin for benign etiologies such as ectopic thymic tissue versus a more malignant pathology. However, it is in the authors' opinion that it should not be routinely utilized but reserved for cases when conventional imaging such as ultrasound or MRI is inconclusive, that is, conventional imaging is suggestive of a malignant etiology and/or staging for known disease.
